# Syndecan-4 as a Marker of Endothelial Dysfunction in Patients with Resistant Hypertension

**DOI:** 10.3390/jcm9093051

**Published:** 2020-09-22

**Authors:** Mark Lipphardt, Hassan Dihazi, Jens-Holger Maas, Ann-Kathrin Schäfer, Saskia I. Amlaz, Brian B. Ratliff, Michael J. Koziolek, Manuel Wallbach

**Affiliations:** 1 Department of Nephrology and Rheumatology, Göttingen University Medical Center, Georg August University, 37073 Göttingen, Germany; dihazi@med.uni-goettingen.de (H.D.); ann-kathrin.schaefer@med.uni-goettingen.de (A.-K.S.); mkoziolek@med.uni-goettingen.de (M.J.K.); manuel.wallbach@med.uni-goettingen.de (M.W.); 2 Department of Transfusion Medicine, Göttingen University Medical Center, Georg August University, 37073 Göttingen, Germany; jens-holger.maas@med.uni-goettingen.de; 3 Department of Cardiology and Pneumology, Göttingen University Medical Center, Georg August University, 37073 Göttingen, Germany; saskia.amlaz@med.uni-goettingen.de; 4 Renal Research Institute and Departments of Medicine, Pharmacology, and Physiology, New York Medical College, Valhalla, NY 10595, USA; Brian_Ratliff@nymc.edu

**Keywords:** Syndecan-4, resistant hypertension, endothelium, baroreflex activation therapy

## Abstract

(1) Background: Arterial hypertension (HTN) is one of the most relevant cardiovascular risk factors. Nowadays multiple pharmaceutical treatment options exist with novel interventional methods (e.g., baroreflex activation therapy (BAT)) as a last resort to treat patients with resistant HTN. Although pathophysiology behind resistant HTN is still not fully understood. There is evidence that selected biomarkers may be involved in the pathophysiology of HTN. (2) Methods: We investigated serum SDC4-levels in patients suffering from resistant HTN before and 6 months after BAT implantation. We collected 19 blood samples from patients with resistant HTN and blood pressure above target and measured serum SDC4-levels. (3) Results: Our results showed high serum SDC4-levels in patients with resistant HTN as compared to a healthy population. Patients with both, resistant HTN and diabetes mellitus type II, demonstrated higher serum SDC4-levels. β-blockers had lowering effects on serum SDC4-levels, whereas calcium channel blockers were associated with higher levels of serum SDC4. BAT implantation did not lead to a significant difference in serum SDC4-levels after 6 months of therapy. (4) Conclusion: Based on our results we propose SDC4 is elevated in patients suffering from resistant HTN. Thus, SDC4 might be a potential marker for endothelial dysfunction in patients with resistant hypertension.

## 1. Introduction

Arterial hypertension (HTN), especially resistant HTN, cause a great amount of damage to the vascular system and enhance the progress of cardiovascular diseases. It is mandatory to treat HTN as early and effectively as possible to prevent further progression to complications like myocardial infarction or the development of chronic kidney disease. Nowadays there are multiple pharmacological treatment options for the treatment of HTN. Patients with ongoing high blood pressure (BP) values despite the appropriate use of more than three antihypertensive drugs (including one diuretic) are categorized as patients suffering from resistant HTN. Baroreflex activation therapy (BAT) is used in those patients as an interventional method to reduce BP. BAT takes its effect mainly through the reduction of sympathetic over activity. Recent studies explain its BP-lowering capability in part due to its effect in increasing natriuresis [1,2]. Meta-analysis confirms the beneficial effect of BAT on the reduction of BP [3]. BAT has also a beneficial effect on the glucose metabolism [4], the arterial stiffness [5] and renal function [6].

An enhanced sympathetic nervous activity is a main component leading to HTN and especially to resistant HTN and its degree of activity was correlated with BP levels and endothelial dysfunction [7,8,9,10,11]. Therefore, it appears obvious that over activity of the sympathetic nervous system could be identified as an important cause of the complex pathogenesis between HTN, chronic heart and kidney diseases which promotes cardio-, renal- and vascular end-organ damage [12,13]. Several biomarkers for HTN have been identified trying to evaluate underlying processes involved in the onset and progression of HTN [14].

Syndecan-4 (SDC4) belongs to a family of proteoglycans, the syndecans, representing one of the components of the endothelial glycocalyx and the basement membrane of endothelial cells [15]. It is the only member of the family with an ubiquitous distribution and is one of the major proinflammatory sensors of endothelial cells [15,16]. In fact, deficiency of SDC4 leads to delayed healing of skin wounds with impaired angiogenesis in the granulation tissue [17], an increased rate of cardiac rupture after myocardial infarction [18] and higher vulnerability to lipopolysaccharide-injections with a considerably higher level of mortality [19]. In addition, promoting endothelial cell alignment, reducing the progress of atherosclerosis and playing a role in cardiac remodeling are other major actions of SDC4 [20,21]. As part of the endothelial glycocalyx the role of SDC4 in angiogenesis has been investigated in multiple studies with heterogeneous results. On the one hand, SDC4 has been linked to pro-angiogenic potential via mediating prostaglandin E2 through the protein kinase Cα [22], or as a receptor for the pro-angiogenic N-terminus of thrombospondin-1 [23]. On the other hand, it has been demonstrated to counteract the effects of endothelial nitric oxide synthase (eNOS), linking it to an anti-angiogenic potential [24]. Since SDC4 is a transmembrane protein it is crucial to associate its effects to the whole molecule or to the separate domains, respectively [25,26]. It is argued that many of SDC4’s harmful effects are linked to its extracellular domain rather than the whole SDC4 molecule itself [27].

Due to its main location on the endothelial glycocalyx, SDC4 is affected by diseases exerting its main damage on the endothelium, like HTN and might serve as a predictor of endothelial dysfunction. Through its damaging effects to the endothelium, HTN results in endothelial dysfunction leading to atherosclerosis and further complications. In fact, sympathetic over activity, which is seen in patients with resistant HTN, is linked to endothelial dysfunction and can be reversed by autonomic blockade [28]. The aim of this study was to investigate whether patients with resistant hypertension had higher levels of SDC4 and to analyze if BAT, as a sympathicoinhibitory intervention, has an impact on SDC4-levels. To the best of our knowledge, SDC4 was not evaluated in patients with resistant HTN so far.

## 2. Methods

### 2.1. Patients, Baroreflex Activation Therapy (BAT) and Study Protocol

Patients with resistant HTN [29] and BP above target (European Society of Hypertension/European Society of Cardiology Guidelines [30,31]) despite intake of at least three antihypertensive drugs including a diuretic, optimization of life-style behaviors and therapy for secondary reasons, were consecutively included into this observational study. The present study presents a retrospective analysis of prospectively collected data from patients with resistant hypertension receiving BAT between 2012 and January 2015 and a control cohort. For BAT, the Barostim neo^TM^ (CVRx, Minneapolis, MN, USA) was used as described previously [32,33]. The lead of the BAT is sutured directly onto the carotid sinus and the pulse generator is implanted in an infraclavicular position in a minimally invasive procedure that includes intraoperative testing for optimal lead placement for BP response [32,33]. BAT was initiated 4 weeks after implant and the stimulation was individually increased by adaption of programmed parameters during monthly follow-up visits. Study visits, which included collecting blood samples, asking about medication adherence and medical history, performing a physical examination, reviewing medication and assessing vital signs, were performed before and 6 months after BAT activation. Modification of antihypertensive medication by the treating physician based on the individual office and/or self-measured BP was allowed during the observation period. In particular, antihypertensive medication was reduced if ≥1 of the following criteria was fulfilled: (1) BP was below individual target, (2) BP was above target and severe symptoms associated with BP reduction (e.g., dizziness) developed and (3) occurrence of typical side effects (e.g., hyperkalemia using aldosterone antagonist) [34]. All patients provided informed consent before the initiation of protocol-mandated procedures. The study has been carried out according to the Declaration of Helsinki and was approved by the local Ethical Committee of Goettingen (19/9/11).

### 2.2. Patient Collective of the Control Group

In order to create a healthy control group, blood samples were taken from men and women who donated blood at the University Medical Center in Goettingen. In this study, donators were included with few to no comorbidities (1 patient suffered from hypothyroidism) and few to no regular intakes of medication (1 patient had a regular intake of levothyroxine) at the time when their blood was taken. Each donator gave their written consent and the collection was approved by the local Ethical Committee of Goettingen (19/9/11).

### 2.3. Office Blood Pressure (BP) and 24 h Measurements

Brachial BP of the arm was recorded after 10 min of supine rest using a semiautomatic oscillometric device (Bosch + Sohn GmbH (Juningen, Germany)) two times within a 3-min interval. The mean values out of these two measurements were averaged.

Measurements of 24 h ambulatory BP were performed using an oscillometric Spacelabs Model 90,207 Recorder (Spacelabs Healthcare, Nürnberg, Germany) with readings taken every 15 min in daytime and every 30 min at nighttime. Ambulatory blood pressure readings were averaged for 24 h. Patients were assessed while adhering to their usual diurnal activity and nocturnal sleep routine. According to the European Society of Cardiology/European Society of Hypertension guidelines, only recordings with >70% valid measurements were included in the analysis [31].

### 2.4. Enzyme-Linked Immunosorbent Assay (ELISA) Analysis of Human Syndecan-4 (SDC4)

Human SDC4 in serum probes collected from blood samples from each participant was analyzed using a human SDC4 enzyme-linked immunosorbent assay (ELISA) kit according to protocol (27188, IBL, Gunma, Japan). It is noteworthy to mention that the ELISA kit targets the ectodomain of SDC4.

### 2.5. Measurement of Pulse Wave Velocity (PWV)

Measurement of pulse wave velocity (PWV) was performed as described before using the SphygmoCor device (version 7.0; Atcor Medical, Sydney, Australia) [5]. In one patient PWV was measured with the Mobil-O-Graph (IEM GmbH, Stolberg, Germany).

### 2.6. Statistical Analysis

Values are given as means ± standard deviation (SD) unless otherwise mentioned. Data were analyzed using a *t*-test, dependent or independent where appropriate, Fisher exact test or analysis of variance (ANOVA). The Spearman correlation coefficient was used to describe the relationship between two metric variables. General linear model was used to adjust for co-variables.

*p* values of <0.05 were considered statistically significant. All statistical analyses were performed with GraphPad PRISM 8 (GraphPad Software, Inc., San Diego, California, United States) and Statistica 13 (TIBCO Software Inc., Palo Alto, California, United States).

## 3. Results

### 3.1. Patients and Blood Pressure

In 19 patients with resistant HTN treated with the BAT Neo system, BP measurements were performed before BAT implantation and 6 months after BAT activation. Patients’ mean age was 61 ± 10 years and 47.4% were male. The mean body mass index (BMI) was 32.43 ± 6.62 kg/m^2^ and 12 patients (63.2%) had BMI > 30 kg/m^2^. This cohort included 13 patients (68.4%) with chronic kidney disease (CKD) stage 3 or higher. Diagnosis of CKD was based on glomerular filtration rate (GFR) and albuminuria according to the current Kidney Disease: Improving Global Outcomes (KDIGO) guidelines [35]. Five patients (26%) had a history of renal denervation. Diabetes mellitus (DM) was diagnosed in seven patients (36.8%). The characteristics of the BAT and the control group are summarized in Table 1.

Baseline characteristics differ in respect to age (*p* < 0.01) and BMI (*p* < 0.01), whereas no differences were observed in respect to gender distribution (*p* > 0.99) between the patients with resistant HTN and controls.

One patient refused follow-up ambulatory 24-h BP measurements and in one patient 24-h measurements were obtained after a period of 12 months. Before BAT implantation patients with resistant HTN showed systolic BP of 160 ± 24 mmHg in office and 144 ± 17 mmHg in ambulatory 24 h BP measurement despite taking an average of 6.8 ± 1.4 antihypertensive drugs. After 6 months of BAT systolic BP was reduced to 139 ± 33 mmHg in office (*p* < 0.01), while systolic 24 h BP and number of antihypertensive medication were numerically reduced to 138 ± 26 mmHg (*p* = 0.09) and 6.4 ± 1.6 antihypertensives (*p* = 0.12) without reaching significance.

### 3.2. SDC4 is Elevated in Patients with Resistant Arterial Hypertension (HTN)

**Figure 1:**
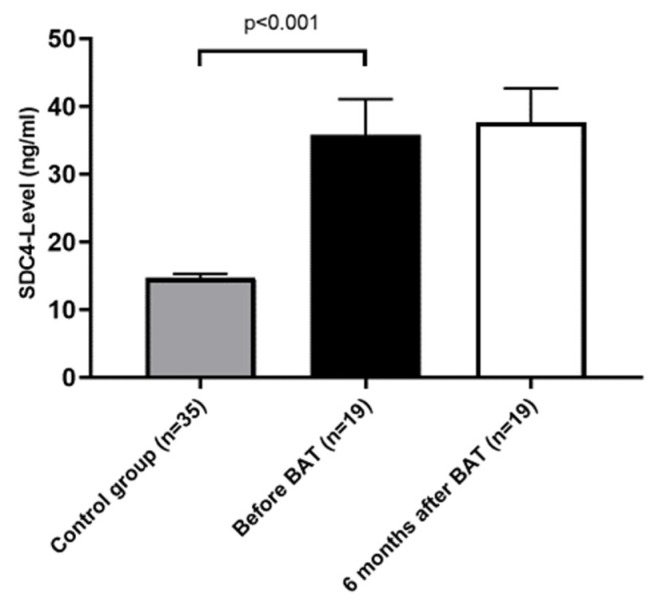
Serum syndecan-4 (SDC4) level is increased in patients with resistant arterial hypertension (HTN). Values are given as means ± standard error of the mean (SEM).

In total, we collected 35 blood samples out of the normal population which represents the control group and 19 blood samples of patients suffering from resistant HTN undergoing BAT. For our group of patients, we collected blood samples on two different time points, just before and 6 months after BAT implantation. We measured a baseline serum SDC4-level of 14.7 ± 0.6 ng/mL in the control group. The serum SDC4-level measured in patients with resistant HTN was significantly higher and reached an average of 35.8 ± 5.3 ng/mL (*p* < 0.001) (Figure 1). Normal values of serum SDC-4 range between 5.7–16.05 ng/mL [36,37,38].

The co-variables age (*p* = 0.42) and BMI (*p* = 0.82) had no significant influence on SDC4-levels. Moreover, differences in SDC4-levels between patients with resistant HTN and controls remained significant (*p* = 0.03), using age and BMI as co-variables. Within the group of patients with resistant HTN SDC4-levels did not differ between patients with or without prior renal denervation (*p* = 0.58) or with or without congestive heart failure (*p* = 0.45), respectively.

Six months after the BAT implantation, another measurement of serum SDC4 revealed an average of 37.7 ± 4.9 ng/mL, reaching no significant difference to the measurement before BAT implantation (*p* = 0.72), indicating BAT to have no effect on the SDC4-level in the serum within the observed 6-month period. Whereas there was no correlation between SDC-4 levels and baseline BP or the level of BP within the cohort of patients with resistant HTN, there was a moderate correlation between systolic 24-h BP change and SDC-4 levels at baseline (r = −0.38, *p* = 0.12) without reaching statistical significance.

PWV is the gold standard for non-invasive measurement of arterial stiffness [39] and reference values are available [40]. A PWV > 10 m/s is considered as an independent cardiovascular risk factor and shows additive value above and beyond traditional risk factors [39,41].

In comparison with an age-matched normotensive reference cohort (PWV 9.4 m/s) [40], the present patients with resistant HTN showed distinctly higher PWV (10.6 ± 3.4 m/s). Whereas PWV was moderately correlated with mean 24 h ambulatory blood pressure (r = 0.34, *p* = 0.17) and age (r = 0.41, *p* = 0.08). There was only a small correlation between PWV and SDC4-levels in patients with resistant HTN in the present study (r = 0.11, *p* = 0.65).

### 3.3. Patients with Diabetes Mellitus (DM) Type II Show Higher Serum SDC4-Levels

**Figure 2:**
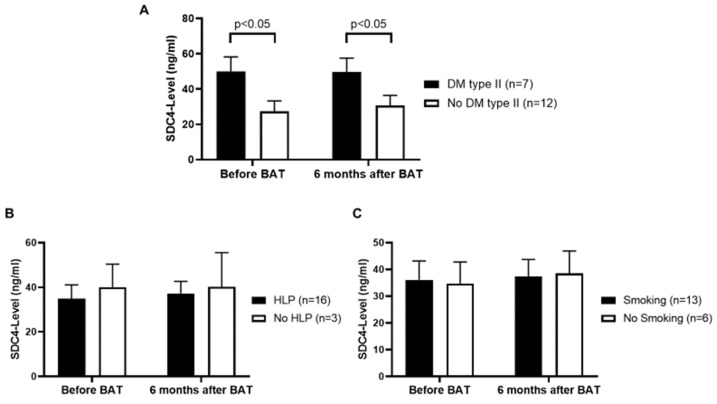
(**A**): Patients who do not suffer from diabetes mellitus (DM) type II show lower levels of serum SDC4 as compared to patients with concomitant DM type II. (**B**): HLP does not show an association with serum SDC4-levels. (**C**): Smoking does not alter serum SDC4-levels. Values are given as means ± SEM.

In order to take a deeper look on the comorbidities of our patient collective comparison of serum SDC4-levels in patients who also suffered from DM type II to those who do not have DM type II was performed. Patients who suffer from both, resistant HTN and DM type II, showed higher levels of serum SDC4 compared to patients without DM type II (*p* < 0.05) (Figure 2A). Patients suffering from resistant HTN with no DM type II still showed higher levels of serum SDC4 as compared to the control group. After 6 months of BAT there was no significant difference in serum SDC4-levels. Comparing serum SDC4-levels in respect to hyperlipoproteinemia (HLP) or smoking status, no significant difference could be detected (Figure 2B,C).

### 3.4. No Influence of Chronic Kidney Disease (CKD) on Serum SDC4-Levels

**Figure 3:**
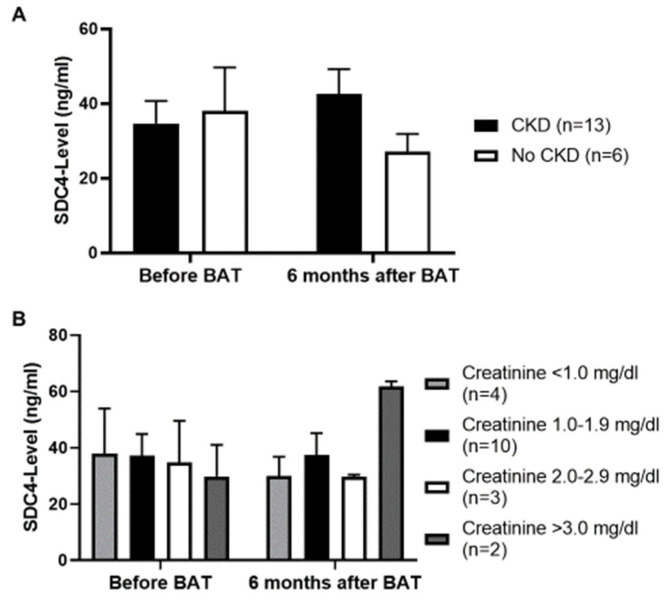
(**A**): No significant difference in serum SDC4-level regarding CKD. However, 6 months after baroreflex activation therapy (BAT) implantation a moderate correlation is seen in patients with no CKD in their history, having lower levels of serum SDC4 as compared to patients with a positive history of CKD (*p* = 0.15). (**B**): The serum creatinine-level does not correlate with the serum SDC4-level. Values are given as means ± SEM.

Based on prior studies [42,43,44], which demonstrated SDC4 to play a key role in the development of tubulointerstitial fibrosis, we compared serum SDC4-levels in patients also suffering from CKD and analyzed serum SDC4-levels based on the respective creatinine level in the serum. Patients with an estimated glomerular filtration rate of <60 mL/min for a period of at least ≥3 months were included in the group of patients with CKD. Before BAT implantation, serum SDC4-levels were equal amongst patients with and without CKD; 6 months after BAT implantation patients with no CKD in their history showed lower levels of serum SDC4 as compared to patients with a positive history of CKD. However, this difference did not reach statistical significance (*p* = 0.15) (Figure 3A). Analyzing the serum creatinine level (mg/dL) we did not detect a significant difference on the level of serum SDC4, although after 6 months of BAT two patients, one of them a dialysis patient, with a creatinine level of greater than 3.0 mg/dL showed quite high levels of SDC4 (Figure 3B).

**Figure 4:**
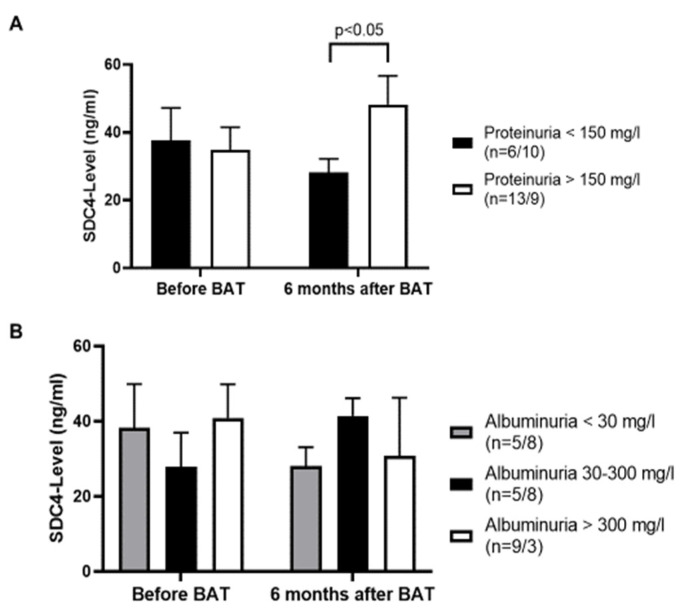
(**A**): Six months after BAT implantation patients with a proteinuria >150 mg/L show higher levels of serum SDC4. (**B**): Serum SDC4-levels do not correspond to albuminuria of patients suffering from resistant HTN. First number of *n* represents patient number before BAT. Second number of *n* represents patient number 6 months after BAT implantation. Values are given as means ± SEM.

In a next step we compared the amount of proteinuria with the measured serum SDC4-level. Interestingly we detected higher serum SDC4-levels in patients with a proteinuria >150 mg/L 6 months after BAT implantation (*p <* 0.05) (Figure 4A). Before BAT implantation the levels of serum SDC4 were almost equal between patients with or without proteinuria. Comparing serum SDC4-levels with the amount of albuminuria we did not find any significant differences (Figure 4B).

### 3.5. β-Blockers and Thiazide Diuretics Decrease, and Calcium Channel Blockers Increase the Serum Level of SDC4

**Figure 5:**
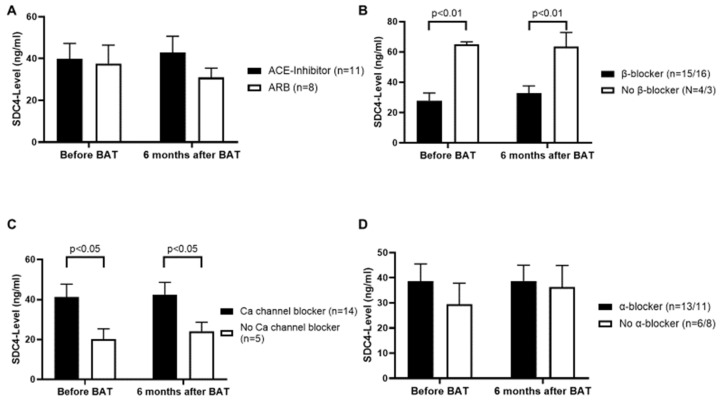
(**A**): Both angiotensin-converting enzyme (ACE)-inhibitors and angiotensin receptor blockers (ARBs) show no correlation to serum SDC4-levels. (**B**): With no β-blocker treatment SDC4-levels are significantly higher as compared to treatment with β-blockers. (**C**): Treatment with calcium channel blockers is associated with increased serum SDC4-levels. (**D**): α-blockers do not alter serum SDC4-levels. The first number of *n* represents patient numbers before BAT. Second number of *n* represents patient number 6 months after BAT implantation. If there is only one number of *n* mentioned, it means that the number of patients taking that medication has not changed after the 6-month interval. Values are given as means ± SEM.

In a next step we studied the medication list of each patient and compared the most common antihypertensive drugs to the respective serum SDC4-level. The intake of an angiotensin-converting enzyme (ACE)-inhibitor or an angiotensin receptor blocker (ARB) revealed no significant impact on the serum SDC4-level (Figure 5A). Furthermore, our results demonstrated on the one hand high levels of serum SDC4 in patients who did not take a β-blocker (*p <* 0.01) (Figure 5B), possibly indicating β-blockers to have a decreasing effect on the serum levels of SDC4. On the other hand, patients with calcium channel blockers in their medication showed higher levels of serum SDC4 as compared to those patients with no calcium channel blockers in their medication (*p <* 0.05), indicating calcium channel blockers to have a possible negative effect on the serum levels of SDC4 (Figure 5C). 15 patients were prescribed β-blockers before and 16 patients after BAT implantation, whereas 14 patients were prescribed calcium channel blockers before and after BAT implantation. The intake of α-blockers did not manifest a significant influence on serum SDC4-levels (Figure 5D).

**Figure 6:**
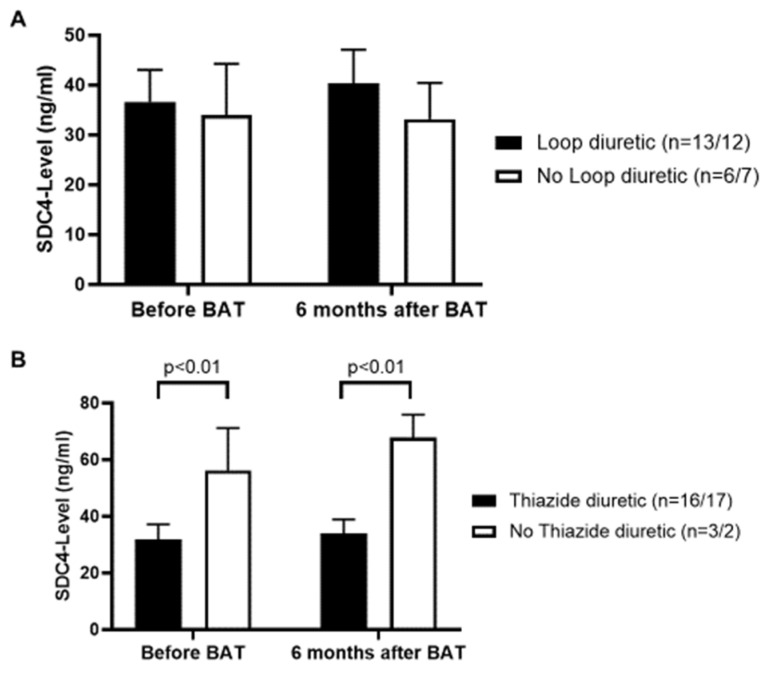
(**A**): Loop diuretics do not correspond to serum SDC4-levels. (**B**): Treatment with thiazide diuretics reveals lower serum SDC4-levels. The first number of *n* represents patient numbers before BAT. Second number of *n* represents patient number 6 months after BAT implantation. Values are given as means ± SEM.

Taking a closer look on therapy with diuretics, loop diuretics could not be established to be of significance regarding the serum level of SDC4 (Figure 6A). However, thiazide diuretics had a possible decreasing influence on the serum level of SDC4, as demonstrated by lower serum SDC4-levels in patients with thiazide diuretics in their medication list (*p <* 0.01) (Figure 6B).

## 4. Discussion

The present study has three major findings: (1) resistant HTN is associated with higher serum levels of SDC4 and (2) β-blockers may have lowering effects whereas intake of calcium channel blockers and the presence of DM type II as a comorbidity may have increasing effects on serum SDC4-levels and (3) BAT exerts no effects on serum SDC4-levels within a 6-month period of treatment.

SDC4 is a transmembrane protein and, therefore, it is important to look at its domains separately in order to understand its signaling properties. In particular, the extracellular domain, also known as ectodomain, is of main interest since the ectodomain acts as a key mediator of cellular adhesion and regulates direct contact of cells with proteins of the extracellular matrix [25,26]. Through the studies of SDC4 it is argued that the harmful effects of SDC4 [17,18,19] are more related to the extracellular ectodomain and not to the whole SDC4 molecule [27]. The ectodomain of SDC4 distinguishes itself from the other two domains of the SDC4 molecule, since it is capable of promoting collagen cross-linking, inducing innate immunity signaling and stimulating immune cell infiltration, consequently being a key mediator in chemotaxis [45,46]. The release of the ectodomain, which involves the process of shedding, is triggered by pro-inflammatory stimuli, like in the event of endothelial injury [47,48], for instance due to HTN.

HTN has been associated with increased levels of matrix metalloproteinase-9 (MMP-9) [49,50] and since SDC4 is a target of MMP-9 [51], HTN leads to the overexpression of MMP-9 to an increased amount of SDC4 shedding, releasing the ectodomain of SDC4 to the circulation and thereby potentially reducing the amount of SDC4 on the endothelial surface, which is suggested to reduce eNOS activity [52]. As preclinical data indicate that endothelial SDC4 knockout results in elevated BP in mice through the decrease of eNOS activity [52], a vicious circle appears possible, in which the elevated circulating SDC4 levels observed in patients with resistant HTN are associated with a decrease in local endothelial SDC4 maintaining the elevated BP in patients with resistant HTN. This pathophysiology corresponds with our results showing patients with resistant HTN to have distinctly higher serum SDC4-levels as compared to the population with no HTN. In addition to that, HTN leads also to oxidative stress [53] which consequently induces metalloproteinase domain-containing protein 17 (ADAM-17) [54], leading to increased shedding of SDC4 [55]. Since the endothelial glycocalyx is the first barrier of the vasculature, it is also the first structure taking damage from HTN, making SDC4 one of the first molecules to disintegrate, suggesting a connection between mechanical sensing, nitric oxide production, and microvascular perfusion. The same mechanisms can be applied to our findings that serum SDC4 reaches higher values in patients suffering from both, resistant HTN and DM type II. MMP-9 and ADAM-17 are also induced in diabetes [56,57]. In addition to that shedding of SDC-4 is increased in inflammatory states which for instance are maintained by resistant HTN and DM type II [58,59].

Based on our results which show increased serum SDC4-levels in patients suffering from resistant HTN the question arises as to whether SDC4 can be considered as a biomarker in those patients. In general, circulating biomarkers in HTN can be divided in markers involved in pathogenesis of HTN, markers indicating HTN progression and markers of cardio-renal-vascular end-organ damage. The group of biomarkers involved in the pathogenesis of HTN consists of markers for endothelial dysfunction, inflammation markers and predictors for the development of HTN [14,60,61]. With HTN as an inducer of shear stress [62] and the knowledge of an upregulated transcriptional expression of SDC4 in the event of shear stress which consequently contributes to the adaptive remodeling of the stressed endothelial glycocalyx [63,64], a causality between SDC4 and HTN seems plausible. However, resistant HTN is a complex scenario with most often already existing end-organ damage and coexisting comorbidities and is highly characterized by sympathetic overdrive, which consequently triggers endothelial dysfunction. Therefore, making a causality between SDC4 and resistant HTN is more delicate and needs a closer look at the vascular system. The condition of the vascular system can be characterized by several non-invasive assessed parameters, such as endothelial-dependent vasodilation, intima-media-thickness, central BP and PWV. Particularly changes in smooth muscle cell and calcification of the vessel’s media lead to elevation of arterial stiffness and thereby increased PWV [65]. The elevated PWV in the presented patients with resistant HTN is above the threshold of 10 m/s, which is an independent risk factor [31]. However, a strong influence of actual BP on PWV without the occurrence of structural vascular damage needs to be considered [5,66]. As SDC4 is part of the endothelial glycocalyx, SDC4 levels might be stronger associated with parameters focussing on endothelial-dependent vasodilatation, e.g., flow-mediated dilatation (FMD), than with parameters of arterial stiffness such as PWV, which depends more on the state of the smooth muscle cells and the degree of media calcification. In accordance with this, there was no significant correlation between SDC4-levels and PWV in patients with resistant HTN in the present study. Techniques evaluating endothelial dysfunction analysing reactive hyperaemia such as FMD or digital vascular response (e.g., EndoPAT) might be favourable for further investigation analysing the role of SDC4.

The role of SDC4 as a biomarker is not unknown. High serum SDC4-levels have been shown in patients with congestive heart failure [27,38] and SDC4 is considered to be a potential new biomarker in those patients. Out of our 19 recruited patients only four were diagnosed with congestive heart failure in which SDC4-levels did not differ compared to patients without congestive heart failure (*p* = 0.45), therefore ensuring our results were not biased by a high number of patients with congestive heart failure. Out of these four patients we measured a mean serum SDC4-level of 43.8 ± 25.2 ng/mL. In patients with congestive heart failure, Takahashi et al. measured in their patient population a serum SDC4-levels of 22.5 ± 12.3 ng/mL [38]. Since only 58% of their patient population were diagnosed with HTN, it is likely that the higher mean serum SDC4-levels (35.8 ± 5.3 ng/mL) in the present patient population are due to resistant HTN, making resistant HTN an aggravating factor for serum SDC4-levels.

Our results also demonstrate a lowering effect of β-blockers on the serum SDC4-level, whereas the intake of calcium channel blockers was associated with higher SDC4-levels. A potential explanation might be that β-blockers are capable of suppressing NF-κB signaling and MMP-9 secretion as reported before [67]. Interestingly, SDC4 is a NF-κB target gene [27], making it even more plausible why β-blockers have a beneficial effect on serum SDC4-levels. In addition to that Haas et al. showed β-blockers to reduce oxidative stress in human coronary artery endothelial cells [68], hence decreasing the activity of ADAM-17, as described before. On the other hand, calcium channel blockers increase for instance the level of MMP-2 with no influence on MMP-9 [69]. Since SDC4 is also a target of MMP-2 [70], it explains our findings of increased serum SDC4-levels in patients treated with a calcium channel blocker. Thiazide diuretics are not well studied regarding possible effects on the process of shedding. Tanner et al. investigated MMP-9 polymorphisms and showed for chlortalidone having suppressing effects on the MMP-9 activity [71].

Of note, the present results neither reveal a correlation between the serum creatinine-level nor the albuminuria and the serum SDC4-level. Based on the current literature [42,43,44] SDC4, and especially its ectodomain, causes increased tubulointerstitial fibrosis. Moreover, SDC4 is known to inhibit RhoA/ROCK activity and consequently to increase TRPC6 gene expression, resulting in increased glomerular permeability [72]. Those findings are substantiated by Kim et al. demonstrating the ectodomain of SDC4 is associated with glomerular pathology [73]. Since SDC4 is highly enriched in endothelial cells of the glomerulus [74] a rise in serum SDC4-levels is more likely during acute kidney injury, rather than in CKD and, therefore, we might see no correlation in our study. Interestingly, our only dialysis patient showed quite high serum SDC-4 levels. The path of SDC-4 degradation is unknown. Based on our finding, one could argue, SDC-4 to be degraded through the kidney. Nevertheless, it would be interesting to investigate serum SDC4-levels in the event of acute kidney injury as well as in a larger cohort of patients with CKD and dialysis patients.

## 5. Limitation

The present study has potential limitations. A major limitation is the small sample size and heterogeneous cohort; however, the influence of differences in age and BMI on SDC4-levels could be excluded. Moreover, Hawthorne effect, lack of randomization and blinding might have affected the present results in respect to follow-up date in patients undergoing BAT. Lack of randomization is the consequence of the current availability of the method as well as ethical reasons to withhold an efficacious therapy from high-risk, well selected patients with resistant HTN.

The fact that our results show no difference in serum SDC4-levels 6 months after BAT implantation, implies that BAT has no influence on the serum SDC4-levels. However, BP values after 6 months of BAT were numerically lower compared to pre-implant data, but still reached an average systolic pressure of 138 mmHg in the 24-h ambulatory BP measurement. Since the endothelial glycocalyx is a very vulnerable structure one can argue that SDC4 is still shed at those BP values. Patients who need an interventional method to reduce their BP might be too chronically sick to take a proteoglycan, SDC4, of a very vulnerable structure, the endothelial glycocalyx, as a marker of BAT implantation success. Considering the localization of SDC4 as part of the endothelial glyocalyx, an early involvement in vascular damage can be derived. Hence, BP reduction through BAT in this advanced stage of HTN with existing end organ damage is presumably too late in the course of this disease to modulate circulating SDC4-levels. The observed moderate negative correlation between baseline SDC4-levels before BAT implantation in patients with resistant HTN with the change in systolic 24 h BP after 6 months might be a starting point for further investigations searching for a prediction marker of BAT response. However, this must be interpreted with caution, as statistical significance was not reached, possibly due to the limited number of participants. It appears at least conceivable that endothelial dysfunction, indicated by elevated serum SDC-4 levels, reflects the degree of sympathetic activity. Further analysis with higher patient numbers and follow-up time would be necessary to make a conclusion, if BAT might influence serum SDC4-levels.

Overall, the present results reveal serum SDC4 to be associated with resistant HTN, especially in combination with DM type II, and with β-blockers to be potentially beneficial lowering serum SDC4-levels. Thus, SDC4 might represent an encouraging biomarker for endothelial dysfunction in patients with resistant hypertension, which needs further evaluation in larger cohorts.

## Figures and Tables

**Table 1:** Characteristics of the control and patient group.

Parameter	Control Group	Resistant Hypertension Group	*p*
*n*	35	19	
**female n (%)** **Male n (%)**	18 (51%) 17 (49%)	10 (53%) 9 (47%)	0.99 0.99
**age (years)**	25.4 ± 3.5	61.1 ± 10	<0.01
**BMI (kg/m^2^)**	25.0 ± 4.9	32.4 ± 6.6	<0.01
**smokers n (%)**	4 (11%)	13 (68%)	<0.01
**relevant concomitant diseases**			
**CKD n (%)**	-	13 (68%)	
**CKD ≥ STAGE 3**	-	12 (63%)	
**DM n (%)**	-	7 (36%)	
**HLP n (%)**	-	16 (84%)	
**CAD n (%)**	-	6 (31%)	
**CHF n (%)**	-	4 (21%)	
**adipositas stage ≥1** **(BMI ≥ 30 kg/m^2^)**	-	12 (63%)	
**number of antihypertensive medications**	0.0 ± 0.0	6.8 ± 1.4	>0.99
**office BP**			
**systolic** **diastolic**	- -	160.2 ± 23.6 81.3 ± 14.9	
**24-H ABP**			
**systolic** **diastolic**	- -	143.1 ± 17.2 76.6 ± 9.9	

Values are mean ± standard deviation (SD) or n (%), body mass index (BMI), chronic kidney disease (CKD), diabetes mellitus type II (DM), hyperlipoproteinemia (HLP), coronary artery disease (CAD), congestive heart failure (CHF), blood pressure (BP), ambulatory blood pressure (ABP).

## Abbreviations

ACE	angiotensin-converting enzyme
ADAM-17	metalloproteinase domain-containing protein 17
ARB	angiotensin receptor blocker
BAT	baroreflex activation therapy
BMI	body mass index
BP	blood pressure
CKD	chronic kidney disease
DM	diabetes mellitus
eNOS	endothelial nitric oxide synthase
GFR	glomerular filtration rate
HLP	hyperlipoproteinemia
HTN	arterial hypertension
MMP	matrix metalloproteinase
SD	standard deviation
SDC4	syndecan-4
SEM	standard error of the mean
